# Annotations

**Published:** 1909-07-17

**Authors:** 


### Annotations.

### Poisonous Hypnotics.

There comes a period in the popularisation of every hypnotic drug when a knowledge of some of its properties diffuses beyond the members of the medical profession, and its name becomes familiar to large numbers of the lay public. The natural result is a series of fatalities; for even if the claims of manufacturers and advertisers, respecting the superiority of their particular article over all others hitherto produced, were actually realised, the indiscriminate use of excessive doses by those who are totally ignorant of physiology and pharmacology is certain to end in calamities. This stage veronal has now apparently attained. It is widely known as a hypnotic drug which has several advantages, and it is extensively used by patients with or without the consent of their doctor, and often without any pretence at medical supervision at all. The result is an increasing frequency of cases such as that investigated in the Paddington Coroner's Court on July 7, when a verdict of death by misadventure was returned respecting a German gentleman who died after the ingestion of 120 grains of veronal. It was stated that he had previously been known to take 60 grains at a time, a dose four times as great as had been originally ordered by his physician. The latter suggested that the sale of patent medicines of this class should be limited to a single safe dose; it is hard to see how the evil may thus be greatly mitigated, for the determined patient can easily keep accounts open with four different chemists, or, indeed, with fourteen for that matter. We draw attention to this case of veronal poisoning especially, because of the growing number of accidents recently in connection with this drug; even doctors themselves have gone down before it, for it may be remembered that early in the summer a London practitioner succumbed to a fatal dose.

### Abdominal Tuberculosis in Britain.

The strenuous efforts which are being made by a small but energetic band of sanitarians and philanthropists in this country to arouse the nation to a sense of the very serious mortality and morbidity from tuberculosis in Great Britain have more than once been the subject of comment and appreciation in these columns. It is encouraging to notice that opinion abroad, especially in the great English speaking Republic across the Atlantic, is also being favourably impressed by these efforts and their results. At the same time there is evident in several recent editorials in American and German papers a tendency to estimate the frequency of tuberculosis in Britain as very greatly exceeding that elsewhere. Whether this is actually the case is not, perhaps, quite so certain; but whether it is or not, the campaign against this scourge must be carried forward with unabated vigour, and without any slackening until our mortality returns show figures very different from the present. One American writer, Dr. Bovaird, has recently compared the statistics of a number of representative children's hospitals in the United States with those of Great Ormond Street, the Edinburgh Hospital for Sick Children, and the Glasgow Children's Hospital with regard to abdominal tuberculosis. As a result of a careful comparison of the returns for a consecutive period of years, he comes to some startling conclusions, which certainly seem to be warranted by the evidence on which they are based. Abdominal tuberculosis, clinically diagnosable as such, is fifteen times as common in Great Britain as in the United States. This author, when he visited Edinburgh, was taken over the Hospital for Sick Children, and was shown, he says, by Dr. John Thomson more cases of unquestionable abdominal tuberculosis among children in one morning than he had seen in ten years in New York. An examination of post-mortem statistics reveals a disproportion nearly as great in respect to primary intestinal tuberculosis. This is found in fully 20 per cent of fatal cases of tuberculosis in childhood in this country, but only in 3.5 per cent. in the United States; and similar percentages are reported from Germany and France. Dr. Bovaird correlates with these figures the much greater frequency of bovine tuberculosis as found by English observers compared with the researches of Continental and American authors. If anyone needs convincing of the seriousness of the milk problem in this country he can be recommended to ponder these facts, whose application and moral are fairly obvious.

### An Ecclesiastic on Vivisection.

Although we would not willingly fail in respect towards those engaged in the task of promoting morality, yet it must be said that passages in Archdeacon Wilberforce's recent sermon to the Anti-Vivisection Congress seem to us deserving of considerable adverse criticism. A published summary of this deliverance contains the following obviously *ex parte* statement, to characterise it no more harshly. "Evidence was accumulating every day that severe suffering was inflicted upon the human race from the erroneous conclusions produced by experiment on animals." Concerning this it may be remarked, firstly, that not so very long ago an ecclesiastical dignitary of the same name gave vent to some remarkable denunciations of the work of Darwin—work which has just received world wide commemoration; and secondly, that the less unreasonable among anti-vivisectionists must see that, with the leavening of public opinion just setting in as the result of the spirited activity of the Research Defence Society, allegations of this sort are bad tactics. We put the matter thus mildly, believing fully in the maxim that in controversy there is nothing so deadly as the strictest moderation. For indeed, perusal of this homily gives one just about as favourable an opinion of the clerical mind as does, say, perusal of *Barchester Towers*—if by quite different means. Archdeacon Wilberforce predicts that in ten years' time it will be clearly recognised that the golden rule of the medical profession is prevention of disease, and not destruction. In the meantime it would be interesting to learn from the anti-vivisection party how, except by animal experimentation, Koch could have conceived the order to stop the watering carts in Hamburg, and thereby stay a fatal cholera epidemic there.

